# Photography in Surgery

**Published:** 1888-04

**Authors:** 


### Photography in Surgery.

We desire to call the attention of physicians generally to the claims which photography has upon them, not because it affords an agreeable, interesting and instructive recreation from routine professional duties, but more particularly because by its cultivation and patronage they will derive the greatest possible aid from it in practice. And, furthermore, it will enable them as a body to collect within a short space of time an amount of scientific *data* otherwise unattainable, which will shed a flood of light upon many mooted points, sufficient to silence discussion forever. Any one who has given the subject the slightest attention will admit without argument that in all pathological conditions which affect the locomotor apparatus, the attitudes which the patients assumes when at rest or in motion differ widely from those which in health are assumed during periods of repose or activity. This fact is admitted by all, but when it comes to give any thing like an accurate and intelligible description of the variation between the attitudes of health and disease, unanimity of opinion is at an end. Even with respect to attitudes of repose there is, as yet, an insufficient amount of available information to render it possible for anyone to give an accurate description of the characteristic attitudes of the various pathological conditions which come under the observations of the surgeon. Nor will it be possible to accomplish this most desirable end until a large number of photographs representative of the class of cases to which this method of observation is applicable have been collected, compared and carefully studied.

If this statenent be true as regards the attitudes in repose, it becomes all the more apparent with regard to locomotive attitudes. For, when it is remembered that in ordinary walking a step is taken every second and sometimes more frequently, and that with each successive step there are movements of the head, arms, forearms, shoulders, trunk and lower extremities, and that some of these—perhaps all of them—differ more or less from the movements in health and are therefore characteristic of the peculiar disability fron which the patient suffers; we say, when these facts are appreciated, the adoption of some method of study and observation which will enable us to come into the possession of definite knowledge regarding the details of these movements, becomes of paramount importance. For the most practised and skillful observer cannot stand by and tell just what happens or how and when certain things are done. It is only by means of photography and the graphic method that such results are obtainable. But to accomplish all this—to formulate and carry out a series of experiments whereby a faithful record of the attitudes, oscillations and movements of the human body in health and disease during locomotion might be made—would require, in addition to ample clinical material, specially constructed places in which to conduct the experiments, specially trained photographic operatives and specially trained scientific workers, all of which means an outlay of a very large sum of money.

But much valuable information can be gathered by individual effort and with limited resources. Every physician finds among his patients illustrations of pathological conditions and attitudes which if accurately photographed, studied and described, would add greatly to his store of useful knowledge, and, when published, to the general store of knowledge upon which an advancement in medical science in certain directions, is dependent.

The attitudes of patients affected with vertebral caries and joint diseases generally, are so characteristic at a very early period in the clinical history of these ailments, that the expert observer can frequently make a positive diagnosis by these means alone. In spinal caries, usually months before there is any angular deformity, the attitude of the patient is perfectly characteristic of the existence of this disease, and not only of its existence but of the particular locality of the spine which is affected.

By the aid of a well-classified collection of photographs, in connection with the study of a few typical living examples, illustrative of the characteristic attitudes of patients affected with spinal caries in different parts of the column, the information which it would take years of independent study to gather can be acquired in a very short time. This statement also holds good with regard to all joint diseases as well as of dislocations, fractures, aneurisms and the various benign and malignant tumors of the hard and soft parts. It will thus be seen that a wide field is open to practitioners everywhere for work in this direction.

Not only should the surgeon photograph the patients when standing, but also in the sitting or recumbent posture as well. It should also be his endeavor in making photographs to select those attitudes which are the most characteristic, and to make the picture from a point of view which will show as much of that characteristic attitude as possible. By so doing much expense will be saved in the reproduction of the photographs for publication.

Though it is not possible to photograph the *locomotor attitudes* of health and disease without an elaborate plant, much useful information can be acquired by the surgeon through the careful observation of them in patients during periods of repose and activity. Probably there is no class of men who have their eyes so well trained in the detection of abnormal locomotor attitudes as horsemen, for by this means alone they are able to recognize, by the gait of the horse--the limp--very readily not only the joint which is affected but oftentimes the character of the lesion. Would that the significance of certain limps of human beings, examples of which we are accustomed to see almost daily, were as generally known among physicians as are the limps of horses among horsemen.

---

When it is a question of nerves, the power of imagination is supposed to be stronger in women than in men, but this was not shown in a recent hospital experiment. Dr. Durand, wishing to test the practical effect of mind disease, gave a hundred patients a dose of sweetened water. Fifteen minutes after, entering apparently in great excitement, he announced that he had, by mistake, given a powerful emetic, and preparations must be made accordingly. Eighty out of the hundred patients became thoroughly ill, and exhibited the usual result of an emetic; twenty were unaffected. The curious part of it is that, with very few exceptions, the eighty "emeticised" subjects were men, while the strong-nerved few, who were not to be caught with chaff, were women.

